# COVID‐19 Mortality and Temporal Trends in a Mid‐Sized Southern Brazilian City (2020–2023): A Population‐Based Retrospective Study

**DOI:** 10.1002/hsr2.72644

**Published:** 2026-06-16

**Authors:** Clodoaldo Antônio De Sá, Junir Antonio Lutinski, Sinval Adalberto Rodrigues‐Junior, Samuel Spiegelberg Zuge, Jeana Cristina Barretta, Felipe Corbellini, Ubiratan Alegransi Bones, João Ventura‐Silva, Vanessa da Silva Corralo

**Affiliations:** ^1^ Universidade Comunitária de Região de Chapecó Chapecó Brazil; ^2^ Escola Superior de Saúde Norte da Cruz Vermelha Portuguesa Oliveira de Azeméis Portugal

**Keywords:** Brazil, Covid‐19, epidemiology, mortality, time factors

## Abstract

**Background and Aims:**

The COVID‐19 pandemic has significantly impacted global mortality, with marked variations according to demographic and temporal factors. This study aimed to analyze COVID‐19 mortality trends and associated demographic factors in Chapecó, Southern Brazil, between 2020 and 2023.

**Methods:**

This population‐based retrospective observational study included all confirmed COVID‐19 cases and related deaths recorded from March 2020 to March 2023. Data were obtained from the Municipal Epidemiological Surveillance Service. Demographic variables (age, sex, and year of occurrence) were analyzed. Mortality was evaluated using multivariable logistic regression models, incorporating variables selected based on epidemiological relevance. Odds ratios (ORs) and 95% confidence intervals (CIs) were estimated. Statistical significance was set at *α* = 0.05.

**Results:**

A total of 84,814 COVID‐19 cases and 861 deaths were recorded (case fatality rate: 1.02%). Mortality was higher among males (61.1%) and increased significantly with age, with a 10.2% higher odds of death per additional year of age. Compared with 2020, mortality risk was higher in 2021 (OR = 2.07; 95% CI: 1.72–2.50) and significantly lower in 2022 (OR = 0.15; 95% CI: 0.11–0.21). Peaks in case incidence during 2022 were not accompanied by proportional increases in mortality.

**Conclusion:**

COVID‐19 mortality in Chapecó was strongly associated with age and male sex. The substantial reduction in mortality after 2021 highlights the impact of vaccination and improved public health measures in mitigating pandemic outcomes.

## Summary

- What is known
  ◦ COVID‐19 mortality increases with age and is higher in men. Brazil experienced its highest death rates in 2020–2021, with reductions following mass vaccination.
- What is new
  ◦ This population‐based study in a medium‐sized Brazilian city shows a 10.2% higher risk of death per additional year of age and a sharp mortality decline after 2021, reflecting vaccination impact.
- Clinical implications
  ◦ Findings highlight the effectiveness of vaccination and the need for sustained surveillance and targeted protection of older adults and men to reduce future epidemic mortality.

## 1 Introduction

The COVID‐19 pandemic has been one of the most impactful public health crises of the 21st century. First reported in Wuhan, China, in December 2019, the disease caused by SARS‐CoV‐2 rapidly spread worldwide and was declared a pandemic by the World Health Organization (WHO) on March 11, 2020. Globally, mortality was strongly influenced by age, sex, comorbidities, and access to healthcare services, with older adults and men at the highest risk of severe outcomes [1, 2, 3].

Brazil became one of the countries most severely affected by the COVID‐19 pandemic. The first confirmed case was reported in São Paulo in February 2020, and the country quickly emerged as a global hotspot for infections and deaths [4]. By September 23, 2023, Brazil had reported 37,796,956 confirmed cases and 705,755 deaths, corresponding to a mortality rate of 347.5 per 100,000 inhabitants [5].

In Brazil, the highest concentration of COVID‐19 cases and deaths occurred in 2020 and 2021, 7,675,973 cases and 194,949 deaths in 2020 (mortality rate: 96.77 per 100,000) and 22,287,521 cases and 619,056 deaths in 2021 (mortality rate: 207.85 per 100,000), whereas in 2022, despite a cumulative 36,331,281 cases, the mortality rate dropped markedly to 35.57 per 100,000 inhabitants [5].

While studies from large urban centers have elucidated pandemic dynamics, medium‐sized cities like Chapecó remain underexplored, despite their unique demographic and healthcare characteristics. Understanding mortality trends in such settings is essential for guiding future epidemic preparedness and local health policies. This study aims to analyze COVID‐19 mortality patterns and associated demographic factors in Chapecó, a mid‐sized city in Southern Brazil, over the period 2020–2023.

## 2 Methods

This population‐based retrospective epidemiological study was conducted using COVID‐19 surveillance data from Chapecó, Santa Catarina, Southern Brazil [6]. Chapecó is the largest municipality in the western region of Santa Catarina, with a population of 254,781 inhabitants, a demographic density of 407.75 inhabitants/km^2^, a schooling rate of 98.4%, a per capita Gross Domestic Product of R$ 53,365.35, and a Human Development Index of 0.790 [7].

During the pandemic, the Municipal Epidemiological Surveillance Service compiled a data set of all confirmed COVID‐19 cases and deaths. All laboratory‐confirmed COVID‐19 cases reported in the municipality between March 2020 and March 2023 were eligible for inclusion in the study. Cases without information on the final outcome were excluded from the analysis. The database included sex, age, date of symptom onset, date of first healthcare visit, reported symptoms, and final outcome (recovery or death). Data were entered into an Excel spreadsheet by a team of municipal health professionals from both the Unified Health System (SUS) and the supplementary healthcare network, who performed daily monitoring and case registration.

This study was approved by the Municipal Health Authority and by the Institutional Ethics Committee for Research Involving Human Subjects (Protocol No. 5.364.429). It followed the ethical principles established in Resolution No. 466/2012 [8] and Resolution No. 510/2016 [9] of the Brazilian National Health Council, as well as the General Personal Data Protection Law. All procedures were conducted in full compliance with the Declaration of Helsinki [10], which governs ethical principles for medical research involving human subjects.

Data were collected by the Municipal Epidemiological Surveillance Service of Chapecó and included the year of registration, the month number since the first reported case (March 2020), age, and sex (male or female). Because the study used the complete surveillance database for the municipality during the study period, all eligible cases were included and no sample size calculation was required. The clinical outcome was recorded as “recovered,” “death from COVID‐19,” or “death from other causes.” For analysis, the outcome variable was converted into a binary indicator, where “death from COVID‐19” was coded as 1 and all other outcomes as 0.

The monthly number of reported cases and COVID‐19–related deaths was used to illustrate the temporal evolution of the epidemic in the municipality. Additionally, monthly incidence rates (cases per 100,000 inhabitants) and case fatality rates (%) were calculated to characterize the local dynamics of the pandemic.

Potential sources of bias include underreporting of cases in the early phase of the pandemic and possible misclassification of causes of death in administrative surveillance records. However, the use of an official municipal epidemiological surveillance database with standardized reporting procedures likely minimized systematic information bias.

Initially, descriptive statistics were used to characterize the study population. Continuous variables were summarized as means and standard deviations, while categorical variables were presented as absolute and relative frequencies.

Multivariable modeling was performed including variables selected a priori based on epidemiological plausibility and existing literature (age, sex, and year of occurrence), rather than relying on automated or *p*‐value–driven selection procedures. This approach was adopted to minimize model instability and avoid bias associated with data‐driven variable selection methods.

Adjusted odds ratios (ORs) and corresponding 95% confidence intervals (CIs) were calculated. Model adequacy was assessed using standard goodness‐of‐fit measures. Statistical significance was defined as a two‐sided *p* value < 0.05.

All analyses were conducted using SPSS version 27.0 (IBM Corp., Armonk, NY, USA).

## 3 Results

A total of 84,814 COVID‐19 cases were reported by the Municipal Epidemiological Surveillance Service of Chapecó from March 2020 to March 2023, with 861 COVID‐19–related deaths, corresponding to a case fatality rate of 1.02%. All reported cases with available outcome data were included in the analysis.

**Figure 1:**
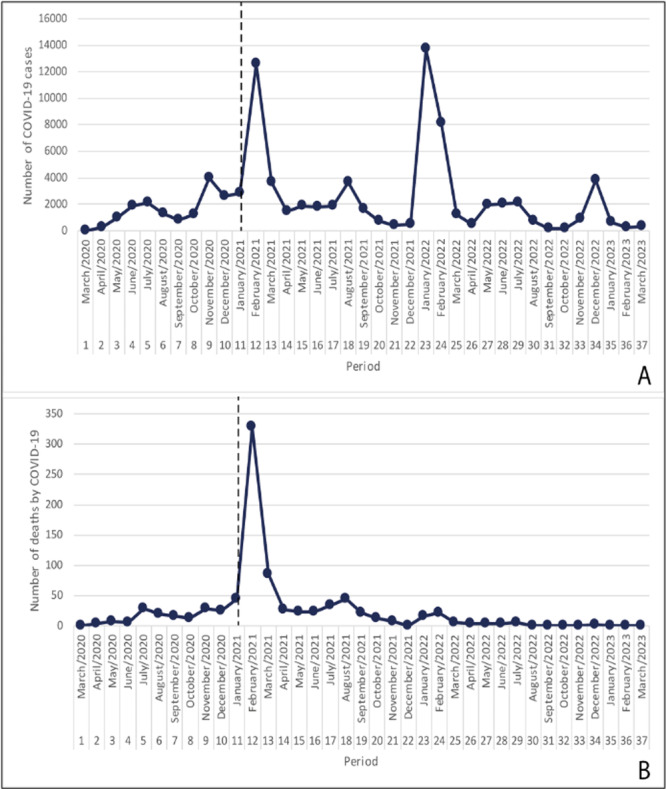
COVID‐19 cases and related deaths in Chapecó, Santa Catarina, Brazil, from March 2020 to March 2023. (A) Number of COVID‐19 cases. (B) Number of COVID‐19 deaths. The dotted vertical line indicates the start of the national vaccination campaign.

The mean age of individuals testing positive was 37 ± 11 years, ranging from less than 1 year to 115 years. Sociodemographic characteristics and the annual distribution of cases are shown in Table 1, and Figure 1 presents the temporal distribution of cases (Figure 1A) and COVID‐19 deaths (Figure 1B).

**Table 1:** Demographic characteristics and annual distribution of COVID‐19 cases (*n* = 84,814) in Chapecó, Santa Catarina, Brazil, from March 2020 to March 2023.

Variables	*n* (%)	95% CI
Year		
2020	15,083 (17.8)	17.5–18.1
2021	33,011 (38.9)	38.6–39.2
2022	35,424 (41.8)	41.5–42.1
2023	1296 (1.5)	1.4–1.6
Age—Mean = 37 ± 11 y		
Age group		
Child	4294 (5.1)	5.0–5.2
Teenage	2832 (3.3)	3.2–3.4
Young adult	32,511 (38.3)	38.0–38.6
Adult	38,919 (45.9)	45.6–46.2
Older	5736 (6.8)	6.6–7.0
Long‐lived older	521 (0.6)	0.5–0.7
Gender		
Male	39,509 (46.6)	46.3–46.9
Female	45,305 (53.4)	53.1–53.7

The first major case peak occurred in early 2021 (January–February) and coincided with the highest mortality peak, with February 2021 alone accounting for 328 deaths (38.1% of all deaths in 2021; Figure 1B). Subsequent case surges, such as those in January and February 2022, did not result in proportional increases in deaths, indicating a decoupling between incidence and mortality likely related to widespread vaccination and improved clinical management.

**Figure 2:**
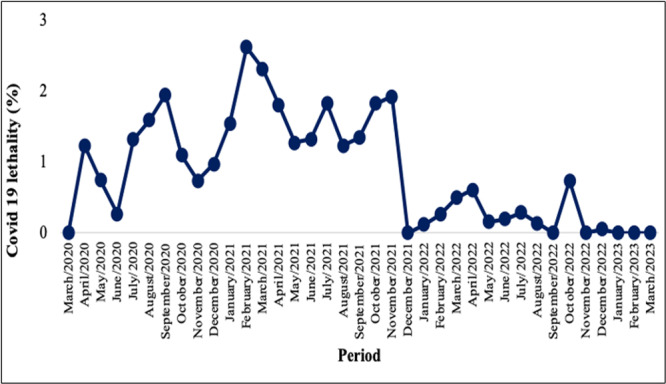
Temporal evolution of the lethality rate of COVID‐19 cases in Chapecó, Santa Catarina, Brazil, from March 2020 to March 2023.

The incidence of COVID‐19 in Chapecó peaked in January 2021 and January 2022, reaching approximately 5000 cases per 100,000 inhabitants (Figure 2). The case fatality rate remained high, near or above 1%, from the onset of the pandemic until November 2021, when a progressive decline was observed (Figure 2). No additional subgroup or sensitivity analyses were performed.

## 4 Discussion

This population‐based study provides a comprehensive evaluation of COVID‐19 mortality dynamics in a mid‐sized municipality in Southern Brazil throughout the full course of the pandemic. The findings demonstrate that mortality was strongly associated with increasing age and male sex (Tables 2 and 3), and that a substantial reduction in deaths occurred after 2021 despite subsequent peaks in case incidence (Figures 1 and 2). These results highlight the importance of demographic risk factors and public health interventions in shaping pandemic outcomes at the local level.

**Table 2:** Bivariate logistic regression analysis of factors associated with COVID‐19–related deaths in Chapecó, Santa Catarina, Brazil, from March 2020 to March 2023.

	Deaths by COVID‐19			
Variable	Yes *n* (%)	No *n* (%)	OR (95% CI)	*p*
Age group				0.001
Child	6 (0.7)	4288 (5.1)	1	
Teenage	0 (0.0)	2832 (3.4)	—	
Young adult	25 (2.9)	32,486 (38.7)	0.55 (0.23–1.34)	
Adult	326 (37.9)	38,593 (46.0)	6.04 (2.69–13.54)	
Older	412 (47.9)	5324 (6.3)	55.30 (24.68–123.94)	
Long‐lived older	92 (10.7)	429 (0.5)	153.26 (66.711–352.10)	
Gender				0.001
Female	335 (38.9)	44,970 (53.6)	1	
Male	526 (61.1)	38,983 (46.4)	1.81 (1.58–2.08)	

**Table 3:** Multivariate logistic regression analysis of factors associated with COVID‐19–related deaths in Chapecó, Santa Catarina, Brazil, from March 2020 to March 2023.

Variable	Deaths by COVID‐19		
	OR	OR (95% CI)	*R* ^2^
*Model*			0.023*
Age group			
Child	1	—	
Teenage	—	—	
Young adult	0.57	0.23–1.39	
Adult	6.24	2.78–13.99	
Older	57.93	25.84–129.85	
Long‐lived older	162.87	70.84–374.42	
Gender			
Female	1		
Male	1.93	1.68–2.22	

*R² = coefficient of determination for the logistic regression model. p < 0.05 indicates statistical significance of the overall model.

The highest burden of mortality occurred during early 2021, corresponding to Brazil's second pandemic wave. As illustrated in Figure 1B, the most pronounced peak in COVID‐19–related deaths occurred in February 2021, coinciding with a period characterized nationally by increased hospitalization rates, widespread circulation of variants of concern, and intense pressure on healthcare systems. This pattern is consistent with findings reported for Brazil during the second pandemic wave [11], reinforcing the external validity of the findings and suggesting that the municipality experienced pandemic dynamics comparable to those observed across the country.

From mid‐2021 onward, a clear decoupling between case incidence and mortality became evident. Although several increases in case numbers were observed during 2022 (Figure 1A), these surges were not accompanied by proportional increases in deaths (Figure 1B). This temporal dissociation is also reflected in the progressive reduction in case fatality rates over time (Figure 2). Similar patterns have been reported following the widespread implementation of vaccination programs and improvements in the clinical management of severe COVID‐19 cases [12, 13], which likely contributed to the reduced mortality risk observed in later phases of the pandemic.

Age emerged as the strongest predictor of mortality in this study. Individuals in older age groups showed markedly higher odds of death compared with children and younger adults (Tables 2 and 3), reflecting well‐established biological mechanisms such as immunosenescence and the higher prevalence of chronic comorbidities among older populations. These findings are consistent with previous studies demonstrating the strong association between increasing age and COVID‐19 mortality [14]. In addition, male sex was independently associated with increased mortality risk (Table 2), corroborating findings from studies conducted in different countries that attribute these differences to a combination of biological, immunological, and behavioral factors [15, 16, 17].

The multivariate analysis also demonstrated important temporal differences in mortality risk. Compared with 2020, the odds of death were substantially higher in 2021, whereas 2022 showed a pronounced reduction in mortality risk (Table 3). This pattern likely reflects the transition from the pre‐vaccination and early vaccination phases of the pandemic to a period characterized by broader vaccine coverage and strengthened public health responses. Evidence from Brazilian studies similarly indicates that vaccination contributed significantly to reducing case fatality rates and severe outcomes during later pandemic phases [12, 18].

Despite these advances, the findings reinforce the importance of maintaining robust epidemiological surveillance systems capable of monitoring changes in infection dynamics and identifying high‐risk groups. The strong association between age and mortality observed in this study highlights the continued need for targeted public health strategies aimed at protecting older adults and other vulnerable populations. In this context, sustained vaccination efforts, early detection of outbreaks, and adequate healthcare system preparedness remain essential components for mitigating the impact of future epidemic waves.

Although this study was conducted in a single municipality, the epidemiological patterns observed are consistent with national trends reported in Brazil, suggesting that the findings may be generalizable to other medium‐sized Brazilian cities with similar demographic and healthcare characteristics.

## 5 Conclusion

The time elapsed since the beginning of the pandemic was decisive in reducing the number of deaths despite the peaks in the number of cases, mainly from April 2021 onwards. The prevalence of mortality was extremely high in the different stages of life when compared to children and adolescents (young adults, adults, older adults, and 80 years and older).

The progression of the COVID‐19 pandemic in Chapecó demonstrated that time since the onset of the pandemic was a decisive factor in reducing mortality, despite the occurrence of multiple case peaks, particularly after April 2021. Mortality was disproportionately higher among young adults, adults, older adults, and especially those aged 80 years and above, when compared to children and adolescents. These findings reinforce the importance of vaccination campaigns and targeted public health strategies to protect the most vulnerable populations and mitigate the impact of future epidemic waves.

## 6 Limitations

This study has some limitations inherent to its retrospective observational design and the characteristics of the available data set:

- The multivariate model included only demographic variables (age, sex, and year of occurrence) and did not incorporate individual clinical information, such as comorbidities, vaccination status, hospitalization, or intensive care unit admission, which are determinants of COVID‐19 severity and mortality and may explain the additional variability not captured in this analysis.
- Vaccination status was not available at the individual level in the surveillance data set. Therefore, although the temporal decline in mortality after 2021 is consistent with the expansion of vaccination coverage, causal inferences about vaccine efficacy cannot be made within this observational framework.
- Viral variant data were not directly linked to individual cases. The interpretation of mortality reductions in 2022 as partially associated with the emergence of less virulent variants (e.g., Omicron) is therefore based on the national epidemiological context, rather than genomic confirmation within the municipality.

Furthermore, although the pseudo‐*R* ^2^ of the multivariate model was modest, this is expected in population‐based mortality analyses, where results are influenced by multiple biological and health‐related factors that are not available in administrative data sets. The model's objective was not to develop a predictive clinical score, but to assess independent demographic associations at the population level.

## Author Contributions

**Clodoaldo Antônio De Sá:** project administration, conceptualization, methodology, validation, investigation, supervision, formal analysis, methodology, writing – review and editing. **Junir Antonio Lutinski:** conceptualization, methodology, validation, formal analysis, investigation, supervision, writing – review and editing. **Sinval Adalberto Rodrigues‐Junior:** conceptualization, methodology, validation, formal analysis, investigation, supervision, writing – review and editing. **Samuel Spiegelberg Zuge:** visualization, validation, conceptualization, data curation, writing – review and editing. **Jeana Cristina Barretta:** validation, investigation, visualization, data curation, writing – original draft, visualization. **Felipe Corbellini:** validation, investigation, visualization, data curation, writing – original draft, visualization. **Ubiratan Alegransi Bones:** validation, investigation, visualization, data curation, writing – original draft, visualization. **João Ventura‐Silva:** conceptualization, visualization, data curation, writing – review and editing. **Vanessa da Silva Corralo:** visualization, validation, conceptualization, data curation, writing – review and editing.

## Funding

The authors have nothing to report.

## Disclosure

The corresponding author, Clodoaldo Antônio De Sá, affirms that this manuscript is an honest, accurate, and transparent account of the study being reported; that no important aspects of the study have been omitted; and that any discrepancies from the study as planned (and, if relevant, registered) have been explained.

## Ethics Statement

This study was approved by the Municipal Health Authority and by the Institutional Ethics Committee for Research Involving Human Subjects (Protocol No. 5.364.429). It followed the ethical principles established in Resolution No. 466/2012 and Resolution No. 510/2016 of the Brazilian National Health Council, as well as the General Personal Data Protection Law. All procedures were conducted in full compliance with the Declaration of Helsinki, which governs ethical principles for medical research involving human subjects.

## Conflicts of Interest

The authors declare no conflicts of interest.

## Data Availability

The data from this study will be made available upon reasonable request to the corresponding author.
